# A Novel Interhemispheric Interaction: Modulation of Neuronal Cooperativity in the Visual Areas

**DOI:** 10.1371/journal.pone.0001287

**Published:** 2007-12-12

**Authors:** Cristian Carmeli, Laura Lopez-Aguado, Kerstin E. Schmidt, Oscar De Feo, Giorgio M. Innocenti

**Affiliations:** 1 Laboratory of Nonlinear Systems (LANOS), I & C Schools of Computer and Communication Sciences (IC), Swiss Federal Institute of Technology Lausanne (EPFL), Lausanne, Switzerland; 2 Department of Applied Mathematics, School of Optics, Universidad Complutense de Madrid, Madrid, Spain; 3 Max Planck Institute for Brain Research, Frankfurt am Main, Germany; 4 Department of Microelectronics, University College Cork, Cork, Ireland; 5 Department of Neuroscience, Karolinska Institutet, Stockholm, Sweden; Duke Unviersity, United States of America

## Abstract

**Background:**

The cortical representation of the visual field is split along the vertical midline, with the left and the right hemi-fields projecting to separate hemispheres. Connections between the visual areas of the two hemispheres are abundant near the representation of the visual midline. It was suggested that they re-establish the functional continuity of the visual field by controlling the dynamics of the responses in the two hemispheres.

**Methods/Principal Findings:**

To understand if and how the interactions between the two hemispheres participate in processing visual stimuli, the synchronization of responses to identical or different moving gratings in the two hemi-fields were studied in anesthetized ferrets. The responses were recorded by multiple electrodes in the primary visual areas and the synchronization of local field potentials across the electrodes were analyzed with a recent method derived from dynamical system theory. Inactivating the visual areas of one hemisphere modulated the synchronization of the stimulus-driven activity in the other hemisphere. The modulation was stimulus-specific and was consistent with the fine morphology of callosal axons in particular with the spatio-temporal pattern of activity that axonal geometry can generate.

**Conclusions/Significance:**

These findings describe a new kind of interaction between the cerebral hemispheres and highlight the role of axonal geometry in modulating aspects of cortical dynamics responsible for stimulus detection and/or categorization.

## Introduction

Cortical areas of the two hemispheres interact through commissural pathways, the largest of which is the Corpus Callosum (CC). Surgical section of the CC, in man, divides the functions of the two hemispheres [1], [2], a condition usually described as “two persons sharing the same body”. The question is how the intact CC integrates the functions of the two hemispheres. Some answers came from studies in the primary visual areas, exploiting the fact that each hemisphere receives information from the contralateral half of the visual field. Visual perception, therefore, requires interaction between the two hemispheres, as it is also indicated by the existence of inter-hemispheric connections corresponding to the vertical midline of the visual field [3], [4]. Indeed previous studies in both animals and man showed that callosal connections: i) enhance or depress responses to stimuli presented near the visual-field midline [5]–[9] and ii) synchronize the activity elicited in two hemispheres by identical stimuli presented near the midline [10]–[14].

**Figure 1:**
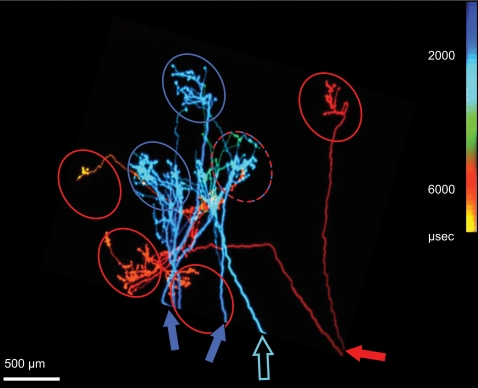
Simulated time of invasion by one action potential (color coded) of three callosal axons. Axons terminating near the border of areas 17 and 18 in the cat, reconstructed from serial sections [15]. Oval-shaped columns selective to horizontal stimuli, schematically redrawn from a separate optical imaging experiment (unpublished) are superposed on the axons. Notice that callosal axons terminate in discrete clusters of synaptic boutons which roughly fit the distribution of the columns. Notice also that action potentials transported by each of the two axons with extensive arbors (filled arrows: for the blue axon two branches are shown whose origin lies outside the figure) reaches the terminal boutons roughly synchronously. However, due to their different thickness, the two axons conduct at different speed, therefore action potentials originating at the same time at their cell bodies arrive at the targets with the considerable delays of roughly 4 ms. The territories of the two axons are partially segregated although they overlap in one of the columns (interrupted red-blue contour). The third axon (open arrow) has a very narrow arbor, and is irrelevant to the present context.

The analysis of histologically reconstructed visual callosal axons suggested the existence of a third mode of interaction between the hemispheres. Individual callosal axons were found to terminate in multiple discrete clusters of synaptic boutons, presumably corresponding to cortical columns [15]. The relative sparseness of synaptic boutons suggested that callosal axons have a modulatory, rather than strongly excitatory “driving” role [16]. Computer simulations indicated that the geometry of most axonal arbors, i.e. the length and thickness of the axonal branches feeding the clusters of terminal boutons of an axon, is appropriate to cause their synchronous activation [15] (Fig. 1). This implies that the individual callosal axons should contribute to generating synchronous neuronal assemblies within the target hemisphere. However, the global effect of the callosal input is not necessarily synchronizing since callosal axons show a large spectrum of diameters, hence of inter-hemispheric conduction delays. At cortical sites where axons with different conduction velocity converge, the callosal input can be desynchronizing. Furthermore, since callosal axons appear to preferentially interconnect cortical columns coding for the same stimulus orientation [5], [11]–[13], [17], the interaction between the two hemispheres should depend on stimulus orientation.

The above predictions attribute an important role in controlling cortical dynamics to a static aspect of brain organization, i.e. axonal geometry. More importantly, assemblies of cooperative cortical neurons implement all brain functions, from perception to cognition. While it seems reasonable to assume that the assemblies are characterized by synchronization of their activity carried out by their interconnectivity this hypothesis has never been rigorously tested for the following reason. Within each hemisphere is extremely difficult to dissociate synchronization of activity due to local or even distant cortico-cortical connections from that generated by re-entrant cortico-thalamo-cortical loops. Furthermore, it might be advantageous to assess synchronization of neural activity using methodologies which are neither restricted to specific frequencies of oscillatory activity, nor to spiking neurons nor to pairs of recording electrodes.

We have tried to achieve the goals mentioned above by studying how inactivating the visual areas in one hemisphere affects the stimulus-driven synchronization of neural activities in the contralateral visual areas. The visual areas of the two hemispheres are interconnected by direct cortico-cortical axons and not by cortico-thalamo-cortical loops. For the study of synchronization we have used a multivariate, frequency independent method derived from dynamical system theory. Finally we have applied the analysis to local field potentials, therefore probing cortical activity at a mesoscopic level, between EEG signal and single neuron recording.

## Results

Synchronization was assessed with a technique derived from dynamical system theory, the S estimator, which measures synchronization by relating it to the shrinking of the embedding dimension of the network of neural oscillators underlying the activity at the different electrode sites [14]. In its simplest formulation S = (1-entropy) and therefore one can say that it estimates synchrony as the degree of order (minimal for S = 0 and maximal for S = 1) in the overall activity across all the electrodes and all the frequency bands. Because of its global nature, the S estimator can be more sensitive than other more frequently employed techniques [14]; we will henceforth call S the value of synchronization it measures.

**Figure 2:**
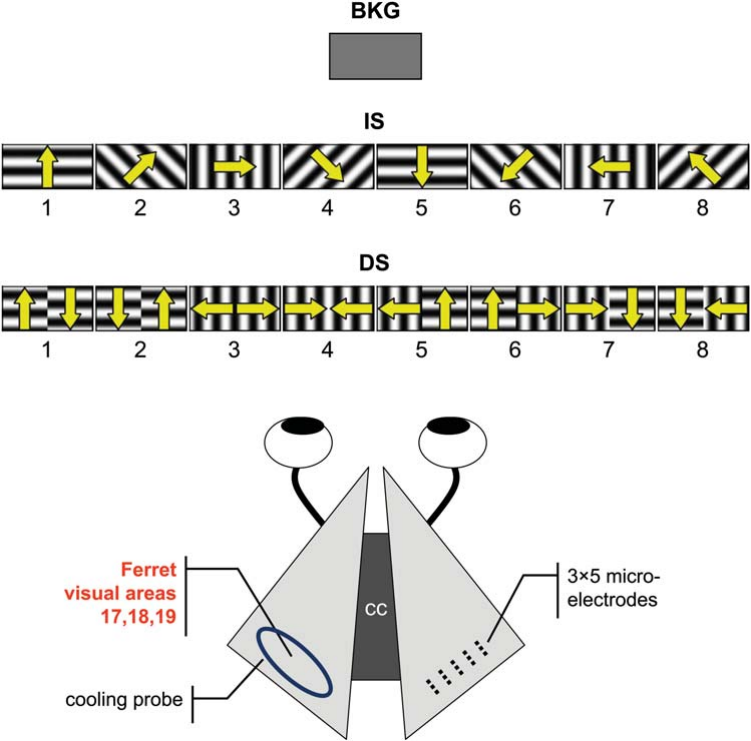
Experimental set up. Anesthetized ferrets viewed either a uniform gray screen (BKG) or gratings moving in the direction shown by the arrows. IS stimuli activated identically and DS differently the hemifields (hence the hemispheres). Local field potentials were recorded with a 3×5 microelectrode array aimed at the visual areas 17, 18 of the right hemisphere. The contra-lateral areas 17 to 19 were reversibly inactivated by cooling.

Three classes of stimuli were presented to anaesthetized ferrets (Fig. 2): i) a homogeneous gray computer screen (background; BKG); ii) 4 gratings oriented around the clock in π/4 rad steps, identical in the two visual hemifields and moving perpendicular to orientation in either direction (the identical stimuli; IS); iii) 8 gratings as above but whose orientation and/or direction of motion differed in the two hemifields (the different stimuli; DS). The two visual hemifields are represented in separate hemispheres and the functional organization of the visual areas is such that BKG and IS activated neuronal pools with identical response properties of two hemispheres while DS did not.

Local field potentials (LFPs) were recorded with an array of tungsten microelectrodes aimed at the strongly callosally-connected representation of the visual-field midline near the border between the visual areas 17 and 18 [4]. We studied how cooling the contra-lateral visual areas affected the intrahemispheric values of S elicited by each stimulus. Seven different microelectrode-array positions were analyzed in 5 experiments, for a total of 14 presentations of BKG and of 56 presentations of both IS and of DS gratings, each repeated 30 times.

Exposure to BKG provided values of S of 0.58, on average (range 0.36–0.71). In all experiments and at all electrode positions the presentation of the gratings significantly decreased the synchronization elicited by BKG (by 18% on average; range 4 to 34%). This was true for 109 of the 112 gratings presented, irrespective of whether they were IS or DS. This grating-induced de-synchronization is not surprising since S = 1-entropy (Materials and Methods) and the gratings carry more information than the BKG stimulus. In other terms, IS and DS, by selectively activating subsets of neurons which specifically respond to the orientation and direction of the gratings, decreased the overall synchrony generated by the homogeneous BKG stimulus over the territory sampled by the microelectrodes.

Cooling the contra-lateral visual areas modified the synchronization elicited by the stimuli.

**Figure 3:**
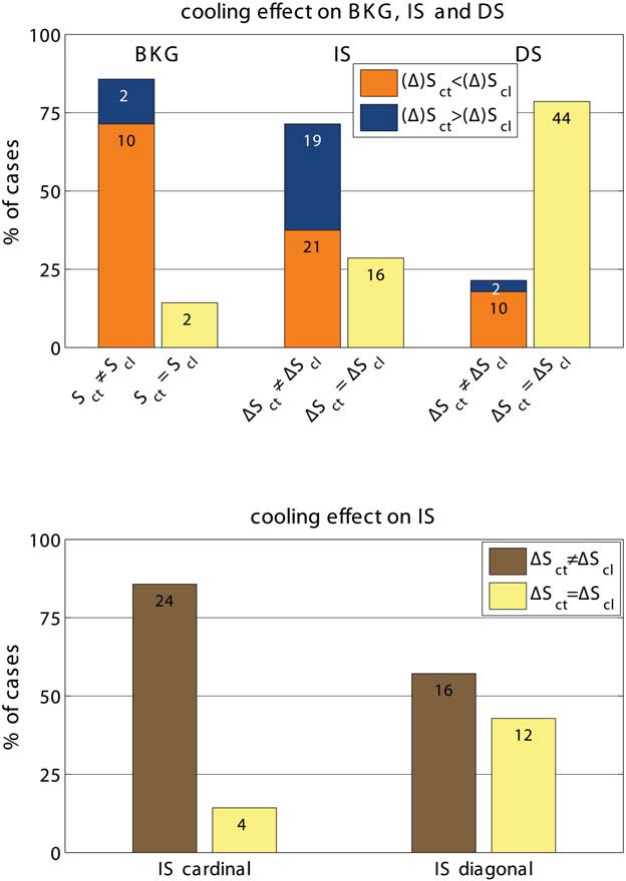
Summary of cooling effects on stimulus-evoked synchronization. Top: Effect of cooling the contra-lateral hemisphere on the value of S during exposure to the background stimulus (BKG) and on that of ΔS (ΔS = S_BKG_-S_stimulus_) during the presentation of the IS and DS gratings. The data are normalized with the number of observations marked on each column segment. Notice that cooling less frequently modified the ΔS elicited by DS than by IS stimuli. The synchronization measured during exposure to BKG appears to be even more frequently affected by cooling. Bottom: Effects of cooling on the ΔS responses to cardinal and diagonal gratings. The responses to the cardinal gratings are more often affected by cooling than those to the diagonal ones. S_ct_ is S during control; S_cl_ is S during cooling; similarly ΔS_ct_ is ΔS during control and ΔS_cl_ is ΔS during cooling.

**Figure 4:**
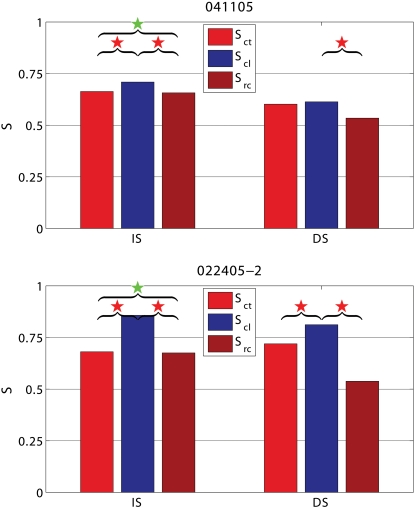
Two examples of the effects on synchronization (S) of cooling and recovery. Consequences of cooling and recovery on S computed during the presentation of BKG interleaved with the IS and DS stimuli in the experiments 041105 and 022405-2. S_ct_ is S in control before cooling; S_cl_ is S in cooling; S_rc_ is S in recovery after cooling. Red stars denote comparisons with statistically significant differences; green stars comparisons with statistically insignificant differences. In both experiments, synchronization during the presentation of BKG interleaved with the IS shows that cooling increases S and that, after cooling S returns to control levels. During the presentation of BKG interleaved with the DS, synchronization is not affected by cooling in experiment 041105 whilst it is increased in experiment 022405-2. Furthermore, after cooling S undershoots control levels in both experiments.

In most cases (10 of 14 presentations) cooling the contra-lateral visual areas significantly increased the synchronization of activity elicited by BKG (by 10% on average; range 2 to 14%). In two cases, cooling decreased the synchronization and in two cases it was ineffective (Fig. 3 top). In all but two cases, interrupting cooling returned synchronization towards control levels or beyond (Fig. 4). Therefore, we concluded that in the presence of BKG the inter-hemispheric input is mainly desynchronizing.

**Figure 5:**
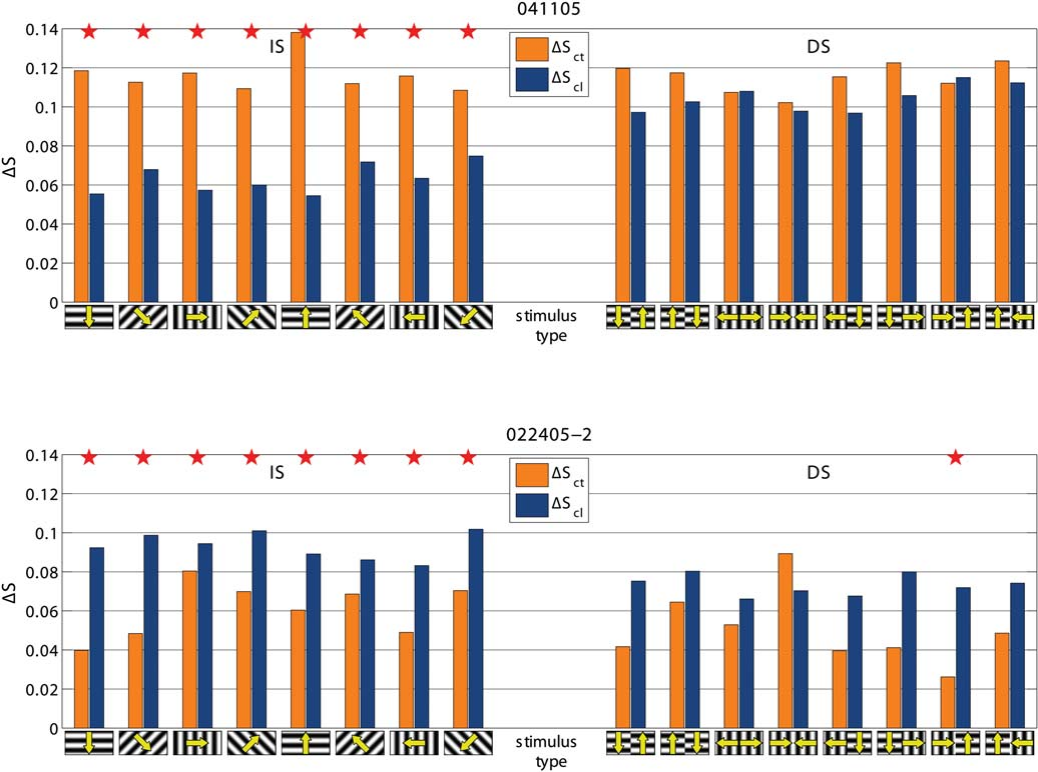
Two examples of stimulus-evoked changes in synchronization (ΔS) in control condition (ΔS_ct_) and during cooling (ΔS_cl_). Statistically significantly different pairs are marked by stars. Notice that cooling affects the responses to IS stimuli, not to DS stimuli. In the experiment 041105 cooling decreases ΔS, i.e. stimulus desynchronizes less (i.e. other hemisphere has a desynchronizing role) and in 022405-2 cooling increases ΔS, i.e. stimulus desynchronizes more (i.e. other hemisphere has a synchronizing role). Other conventions are as in Figs. 2 and 3.

Cooling also modified the de-synchronization caused by the gratings relative to BKG (ΔS = S_BKG_-S_stimulus_). Frequency and statistical significance of the ΔS modulation depended on the type of grating. This is illustrated in the two examples shown in Fig. 5. In the experiment 022405-2 cooling increased the de-synchronization elicited by the grating therefore suggesting a synchronizing effect of the contralateral hemisphere. However the effects were only significant for the IS stimuli. In the experiment 041105, cooling decreased the de-synchronization elicited by the stimulus, therefore indicating a de-synchronizing effect of the contralateral hemisphere. Again significant changes were observed only with the IS, not with the DS gratings. Fig. 3 shows the distribution of the statistically significant cooling effects across the different stimulus presentation. As mentioned the majority of responses during BKG (12 of 14; 85.7%) were affected. So was the majority of the responses to IS gratings (40 of 56; 71.4%) but only 12 of the 56 responses (21.4%) to DS gratings. Furthermore, cooling affected more consistently the ΔS elicited by the vertical and horizontal gratings of IS (24 of 28 cases; 85.7%) than that elicited by the diagonal gratings (16 of 28 cases; 57%; Fig. 3 bottom).

The results described indicate that in presence of the stimulus S had two components: a direct one presumably mediated through the retino-geniculo-cortical pathway and intra-hemispheric connections, and an indirect, inter-hemispheric component, presumably mediated via the CC. The inter-hemispheric component of S depends on stimulus configuration. This suggests that inter-hemispheric interactions can contribute to the early stages of visual cortical processing by modulating in a flexible, stimulus-dependent way temporal parameters of neuronal population activities likely to be involved in stimulus detection and/or categorization [18]–[21].

Cooling either increased or decreased the de-synchronization elicited by IS in almost identical proportions. ΔS increased in 21 of 40, cases (52.5%); it decreased in 19 of 40 cases (47.5%). To understand the cause for this dual synchronizing/de-synchronizing effect, we tested the possibility that the outcomes of cooling may depend on the baseline value of S. However, no relation was found between the changes in ΔS due to cooling and the value of S before cooling. We also double checked the stability of ΔS over time. We first plotted ΔS as function of time for all stimulus presentations in the control and in the cooling condition for the two experiments shown in Fig. 5. All the graphs were consistent, showing that S fluctuated with time around a mean and, when small trends existed, they were consistent in the control and in the cooling conditions. We also divided the control and the cooling epochs into two equal parts and performed the statistical comparison between all the four possible combinations of half epochs. The results were consistent with those obtained with the whole epochs, although in some cases the decrease in the data eliminated the statistical significance. We knew from a separate analysis of the same data (in progress) that cooling either increased or decreased the amplitude of the averaged LFPs recorded at each electrode of the array. Therefore, we tested the possibility that the direction of the ΔS changes depended on whether the amplitude of LFPs at the individual electrodes mainly increased or mainly decreased with cooling. This was also excluded.

Since the synchronization of neuronal pools recorded at one electrode as LFP might be reflected by power spectra we computed the pooled spectra of all responsive electrodes in an exploratory sample of three experiments. This showed that compared to BKG, IS and DS caused power changes below 10 Hz. These changes were affected by cooling. The changes caused by either stimulus or cooling varied across experiments in ways unrelated to those of S.

Instead, the modulation of the stimulus–evoked de-synchronization seemed to depend on the location of the microelectrode-array. ΔS increased with cooling at 3 locations, decreased at 3 other locations and had mixed changes at one. At any given position of the array, ΔS consistently either increased or decreased in repeated IS-stimulation/cooling cycles. The magnitude of the changes in the two directions was almost symmetrical with an average decrease of ΔS of 46% (range 30 to 63%) and an average increase of 50% (range 8 to 132%). In over 70% of cases interrupting cooling returned synchronization towards control levels. Cooling could also either increase or decrease ΔS caused by the DS stimuli, but in most cases the effects did not reach statistical significance.

**Figure 6:**
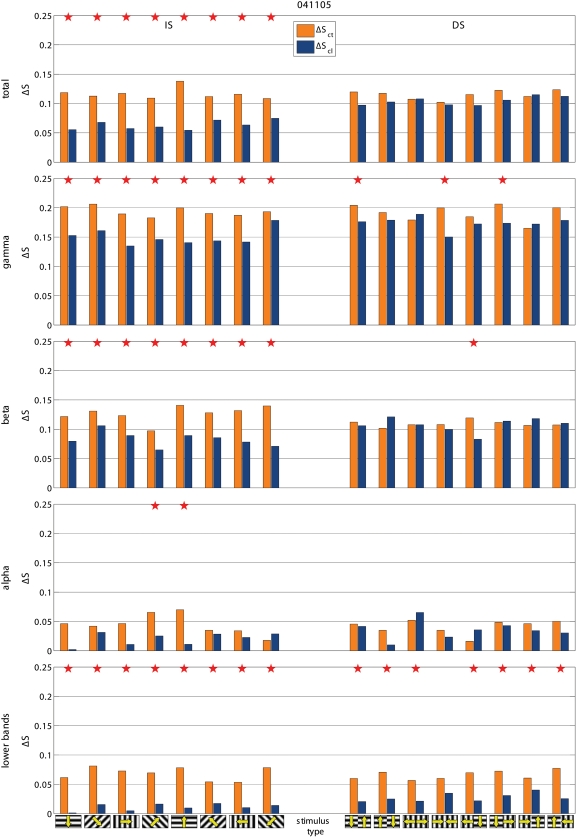
Examples of the effects of cooling on total ΔS and on ΔS calculated for the different frequency bands. Same experiments and conventions as in Fig. 3. Notice that the effects tend to be consistent across the different frequency bands but do not always reach statistical significance.

**Figure 7:**
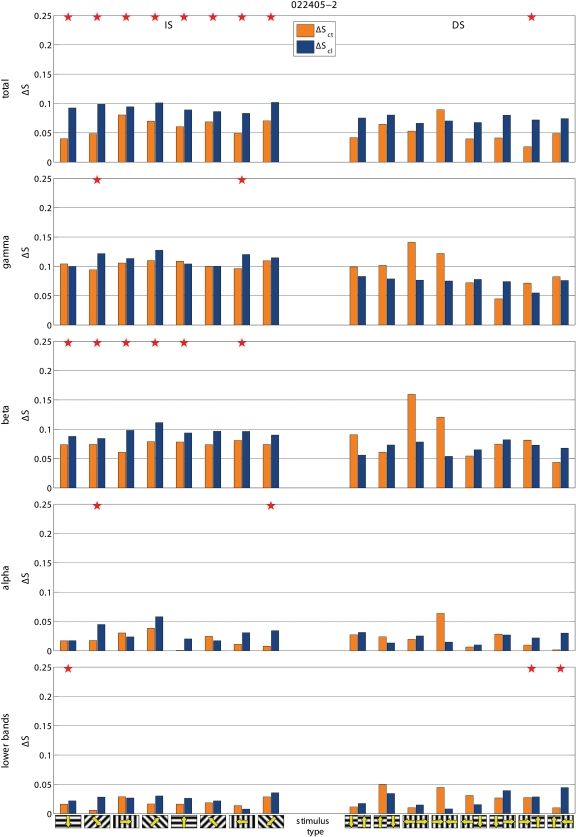
Examples of the effects of cooling on total ΔS and on ΔS calculated for the different frequency bands. Same experiments and conventions as in Fig. 3. Notice that the effects tend to be consistent across the different frequency bands but do not always reach statistical significance.

**Figure 8:**
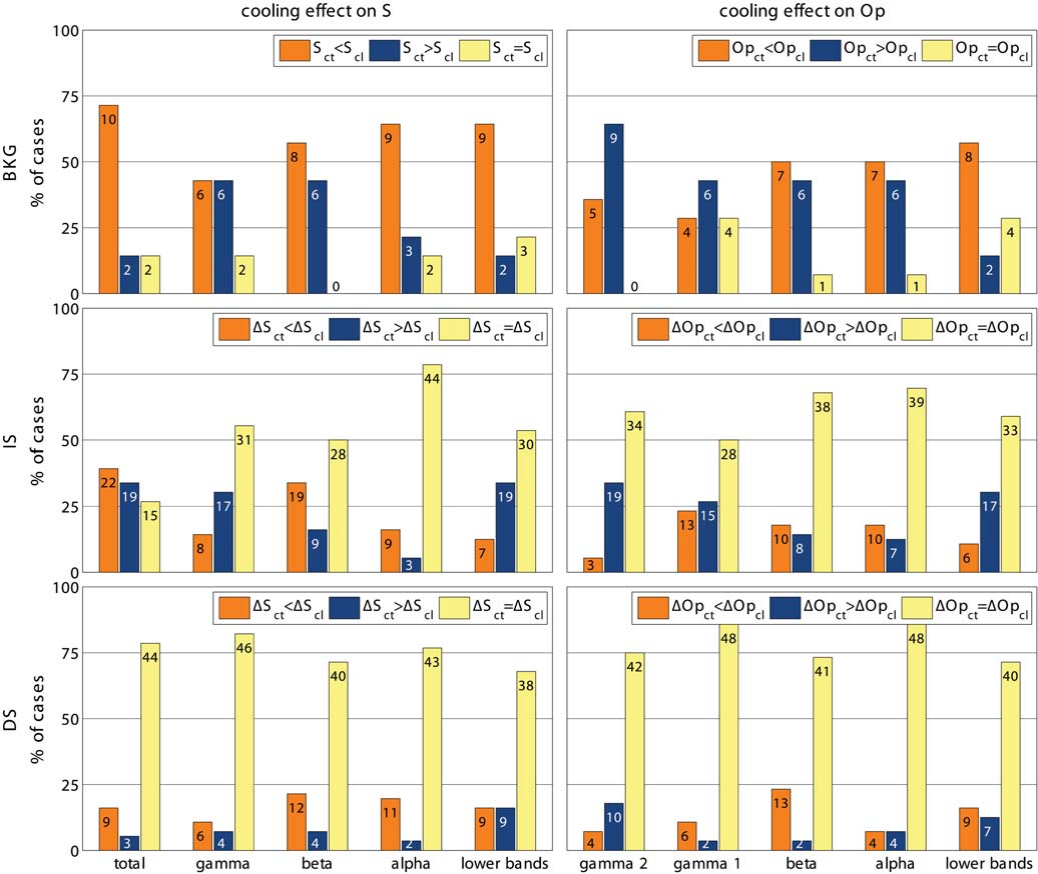
Summary of effects on the different frequency bands for S and for phase synchronization. Conventions as in Fig. 3. Notice that S and phase synchrony return comparable results. In particular, in all bands the most robust effects are obtained during exposure to BKG, followed by the IS stimuli, while there is almost no effect of cooling on the responses to DS stimuli.

To test if the effects on synchronization were specific for a given frequency component of the LFP, we computed S separately for the conventional EEG bands, i.e. gamma (30–70 Hz), beta (13–30 Hz), alpha (7–13 Hz) and lower bands (<7 Hz), as in [14]. We found that the effects of blocking inter-hemispheric cross-talk were stimulus dependent in all frequency bands. S was more frequently affected during the presentation of BKG than of IS and during the presentation of IS than of DS, (Figs. 6, 7, and 8). The effects seemed slightly more robust in the gamma and beta bands, although the lower bands could also be affected and significance of changes in the different bands varied across experiments.

**Figure 9:**
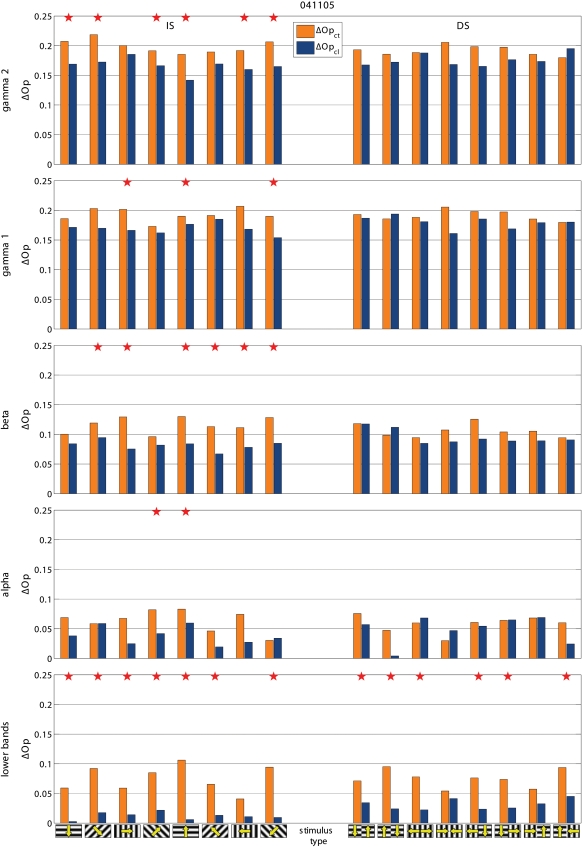
Examples of the effects of cooling on phase synchronization. Same experiments and conventions as in Figs. 3, 6, and 8. Notice that the effects are globally comparable to those obtained with S although they reach less frequently statistical significance. For easier extraction of the phase the gamma band was split into two sub-bands: i.e. gamma 1 (30–50 Hz) and gamma 2 (50–70 Hz).

**Figure 10:**
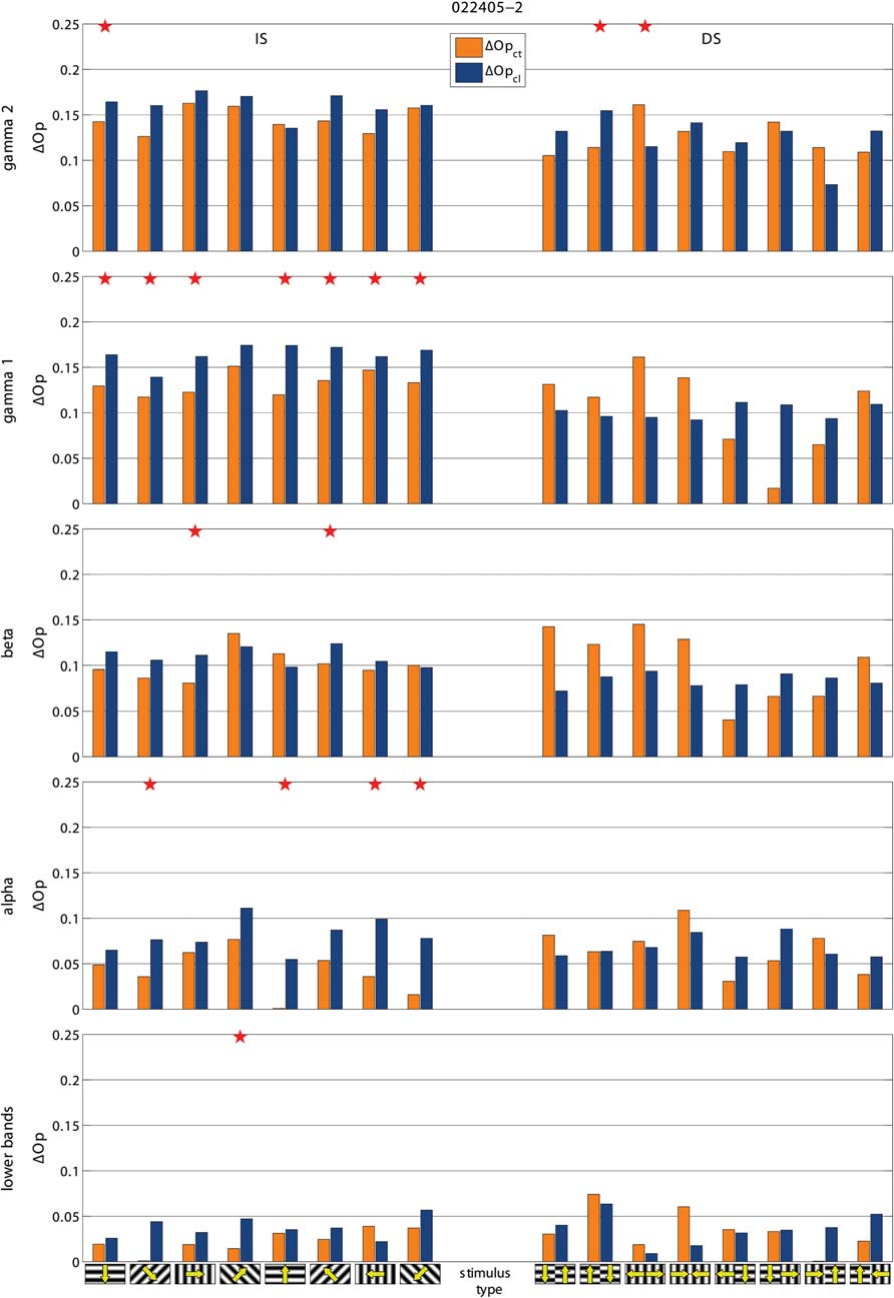
Examples of the effects of cooling on phase synchronization. Same experiments and conventions as in Figs. 3, 6, and 8. Notice that the effects are globally comparable to those obtained with S although they reach less frequently statistical significance. For easier extraction of the phase the gamma band was split into two sub-bands: i.e. gamma 1 (30–50 Hz) and gamma 2 (50–70 Hz).

We wanted to compare the effects obtained with S with those of the frequently used phase synchronization (Materials and Methods and Figs. 9, 10, and 8). The effects resembled those reported above in that the effects of cooling depended on the stimulus, being most robust with BKG than with IS and with IS more than with DS. The gamma and beta bands were also slightly more responsive although the other bands were affected as well.

## Discussion

The method employed in this study for detecting synchronous activity is grounded in dynamical system theory and as such it provides a conceptual approach to neural activity different and in some respect broader than more commonly employed methods to assess cortical synchronization. The main advantages are that the method is multivariate, i.e. it can be applied to the full set of electrodes or it can be broken down to a chosen (≥2) number of electrodes. Second, the method is frequency independent, in line with a rigorous mathematical definition of synchronization [22], [23]. Although synchronization of neural activity, in particular in sensory areas may involve preferentially certain frequency bands no “a priory” arguments dictate that this should always be so. Therefore it can be useful to use frequency-independent methods for detecting synchronization, in particular since, when necessary, the S estimator can be applied to individual frequency bands. Finally in this study and previously the S estimator was found to compare favorably to other methods [14].

**Figure 11:**
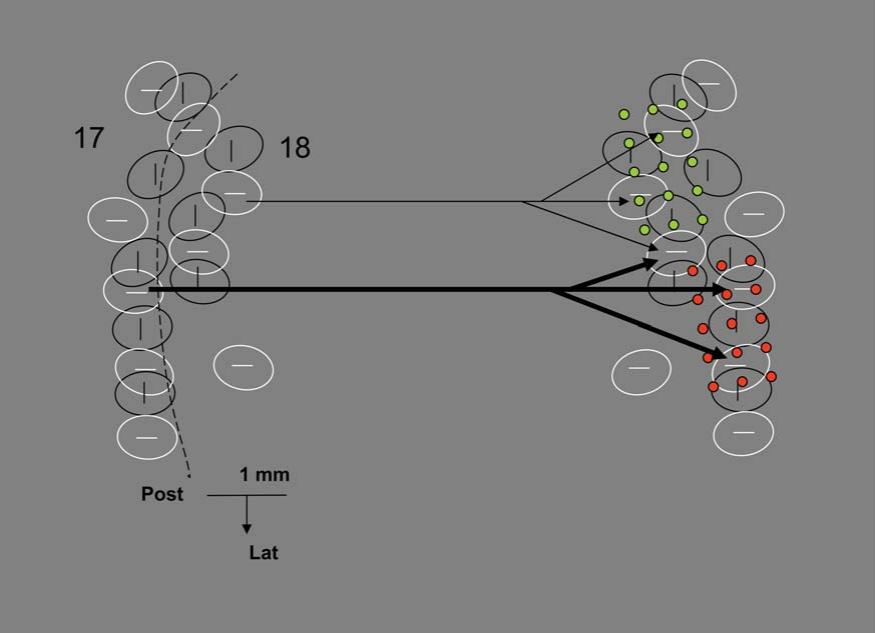
A model of interhemispheric interactions via callosal axons. The figure shows surface views of two sets of orientation columns (vertical and horizontal) in each hemisphere, redrawn and schematized from an optical imaging experiment in the ferret (unpublished). Iso-oriented columns are interconnected as shown by physiological and imaging experiments (text). Two sets of axons of different thickness and hence conduction velocity are shown; each axon diverges to 2–3 columns within a surface of 1 to 1.5 mm^2^ as shown by the reconstruction of single axons in the cat ([15] and Fig. 1). Each axon activates its targets synchronously but differences in conduction velocity among axons cause asynchronous activations of targets reached by the two axons. The model implies a partial spatial separation in the termination of axons of different conduction velocity such that mainly synchronizing effects of contralateral input would be observed at the two electrode arrays positions shown (green and red dots; drawn to scale) while mainly desynchronizing effects would be observed at intermediate locations sampling from the terminations of different conduction velocity axons. A segregation of callosal axons with different conduction velocity was indeed observed in the cat [15]. Which target neurons are contacted by callosal afferents is only partially known. Thalamo-cortical neurons, local inhibitory interneurons as well as, probably, callosally projecting and other cortico-cortical projecting neurons receive callosal input [3], [29], [35], [36].

The effects we described were recorded in parts of the visual areas strongly connected through the CC and were strikingly consistent with the predictions based on structure of CC axons. First, the results confirmed the predicted modulatory, rather than strongly excitatory role of callosal axons in the primary visual areas [16]. Second, the fact that inter-hemispheric modulation of cortical synchrony was more robust with the IS than with the DS gratings is consistent with the proposal that callosal axons, as intra-areal axons [24] preferentially interconnect neurons with the same selectivity for stimulus orientation [5], [11]–[13], [17]. Finally, as shown in the model of Fig. 11, the fact that contralateral input increases local synchrony is consistent with the geometry of callosal terminal arbors, the majority of which appear to impinge onto spatially distributed targets with minimal conduction delays [15]. The desynchronizing effect of inter-hemispheric interactions observed in about 50% of the recording sites could be due to the concurrent activation of axons with different diameters, a general feature of callosal connections [25]–[28], since the different conduction velocities of these axons must lead to temporally disperse activation of their targets. The finding that either synchronizing or desynchronizing effects were reliably observed at a given location of the electrodes, is consistent with the finding that axons with different conduction velocity arrive to partially separated territories ([15] and Fig. 1).

Our findings stress the role of axonal geometry, a static aspect of cortical organization in controlling cortical dynamics in a flexible, stimulus specific way and in, particular, they attribute a role to a striking and usually neglected aspect of cortical organization, i.e. the existence of channels of inter-hemispheric communication of different conduction velocity. In this light, the desynchronizing effects may actually be larger than those predicted by the axonal reconstruction shown in Fig. 1 since conduction velocity differences among callosal axons up to 20 times were measured in electrophysiological experiments (reviewed in [29]) and even larger differences can be predicted by the electron-microscopic data [26], [27].

One interesting aspect of the findings described here is that the formation of synchronous neuronal assemblies in the visual areas is suspected to underlie stimulus detection and/or categorization [18]–[21]. If the formation of such assemblies, in turn depends on axonal geometry, one would expect that in development the latter should be controlled, at least to some extent, by epigenetic information derived from experience. This is precisely what happens in the development of visual callosal connections [30].

## Materials and Methods

Five ferrets bought from a Swedish authorized breeder were prepared for the experiment and anesthetized with intramuscular doses of ketamine hydrochloride (Ketalar 10 mg/kg) and medetomidin hydrochloride (Domitor 0.3 mg/kg), supplemented with atropine sulphate (0.15 mg/kg). After inserting a cannula into the femoral vein and a tracheal tube, the animals were placed in a stereotaxic frame, and maintained on gas anesthesia (1–1.5% isoflurane in 1:1 nitrous oxide and oxygen). The animal was paralyzed with an initial intravenous injection of pancuronium bromide (Pavulon 0.15 mg/kg), supplemented with a continuous infusion of the same drug (6µg/kg/h, 3 mg in 50 ml normal saline, with glucose 10%). The expired CO_2_ was maintained at 3.3–4.0%, body temperature at 37–38°C, and heart rate was constantly monitored. This was performed according to protocols conform to Swedish and European Community guidelines for the care and use of animals in scientific experiments and approved by the ethic committee of Stockholm District (as in [4]). During the experiments they viewed two types of gratings: IS stimuli consisted of 4 full-field gratings oriented around the clock in π/4 rad steps and identical in the two hemifields; DS stimuli were gratings as above but whose orientation and/or direction of motion differed by π/2 rad in the two hemifields. The stimuli remained stationary for 0.5 s and then moved in one of the two directions perpendicular to their orientation for 3 s followed by 3 s of exposure to an equiluminant gray screen, the BKG stimulus. The gratings had a spatial frequency of 18/π cycle/rad and moved at 7π/90 rad/s. Local field potentials (LFPs) were recorded with an array of 3×5 tungsten microelectrodes (two of which permanently inactive) spaced at 410 µm from each other (Frederick Haer, 1.2 MΩ) aimed at areas 17–19. The position of the microelectrodes was controlled histologically after the experiment. Hand mapped receptive fields confirmed by computerized mapping indicated that the electrodes recorded activity within 2π/9 rad from the visual-field midline, i.e. in parts of the visual-field representations which are connected by axons of the corpus callosum [4]. A custom made cryoloop as in [31] was placed on the areas 17–19 of the left hemisphere. The cryoloop was cooled to 2°C +/− 1.5°C as in [7], [31] over 20 min, generating cortical temperatures in the order of about 20°C. This procedure is known to deactivate all cortical layers under the probe. After 5 minutes waiting meant to stabilize the temperature, one stimulation cycle was performed, followed by 30 minutes recovery to normal temperature, after which a new stimulation cycle was performed.

All data were pre-processed in the following way: the 50 Hz power line was removed with a notch filter and the frequency range 0–70 Hz was kept by low-pass filtering. The filtering procedure ensured that no phase lags were introduced. The standard structure of local field potentials recordings over the electrodes was the following: 0.5 s during the presentation of the static IS or DS gratings), 3 s during the presentation of the moving gratings, 3 s when the BKG stimulus was presented. The periods of moving and BKG stimuli were analyzed but the first second of each period was considered a transient, and discarded such that only the last 2 seconds of the stimulation periods were taken for the further processing. Generally, 30 trials were collected for both the BKG and the moving stimulus during control, cooling and recovering after cooling. Noisy or unresponsive electrodes were discarded; on average, 10 electrodes were selected for the further processing. All data were de-trended to zero mean and normalized to unitary variance.

To assess changes in the degree of cooperativeness among the recorded neuronal populations due to the presence of the structured stimulus with or without inter-hemispheric influence, we used the S estimator, that we computed on the whole set of recording electrodes. The S estimator exploits a theoretical consequence of synchronization phenomena to indirectly assess and quantify the synchronization within a set of measurements of arbitral cardinality [14]. Considering a network of dynamical systems, the observable dimensionality (embedding dimension) of the whole dynamical network decreases in consequence of the interactions amid the elements of the networks [22], [23]. For example, when considering a very simple dynamical network consisting of two planar pendula; according to Newtonian mechanics, each one of them has dimension two, given by its position and velocity; by considering them together, the whole network has putative dimension four. However if, the two pendula oscillate together (perfectly synchronized) the “observable” dimensionality of the whole network is only two. In fact, of all the possible four dimensional state combinations (position and velocity of the first and second pendulum) the trajectories of the two synchronized pendula visit only the subpart where the two speeds and two positions are equal to each other, which is a two-dimensional subset of the whole four-dimensional state-space.

The S estimator indirectly measures the synchronization-induced contraction of the embedding dimension by measuring the dispersion (entropy) of the eigenvalues of the correlation matrix of a multivariate set of measurements.

In formulae, given a *P*-variate time series **Y** = {*Y_t_*}, *t* = 0,…,*L*−1, where *Y_t_*∈R*^P^* is the *t*-th sample observation vector and *L* is the number of available samples, and where, without any loss of generality, to make the synchronization measure independent of the relative amplitudes of the signals, we assume that **Y** has been mean de-trended and normalized to unitary variance. For the given time series **Y**, the S estimator is defined as
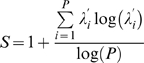
where *λ_i_*′ = *λ_i_*/*P* designate the normalized eigenvalues of the correlation matrix of the multivariate time series **Y**.

By means of its definition, the S estimator quantifies the amount of synchronization within a data set by intrinsically comparing the actual dimensionality of the set with the expected full dimensionality of the asynchronous set.

To understand how the entropy (dispersion) of the eigenvalues of the correlation matrix relates to the dimensionality of the dynamical phenomenon behind the observation is sufficient to resort to one of the main results of linear algebra [32]. In fact, according to the Singular Value Decomposition, the eigen-decomposition of the correlation matrix provides a linearly transformed coordinate system for the original time series **Y**. In this new coordinate system, each normalized eigenvalue gives the relative importance of its corresponding eigen-direction, namely how much this eigen-direction (which is one of the system's dimensions) is visited by the observed trajectory. Consequently, the entropy of the normalized eigenvalues of the correlation matrix accounts for how many dimensions are significantly visited by the observed trajectory. Indeed, when all the normalized eigenvalues are roughly of the same value (maximal dispersion of eigenvalues), all the state-space dimensions are almost equally visited; in this case the entropy of the eigenvalues is maximal (close to 1), consequently S is close to 0, meaning no contraction of the embedding dimension, namely no synchronization. Alternatively, when all the normalized eigenvalues are roughly 0 and only few of them are appreciably nonzero (minimal dispersion), only few state-space dimensions are visited; in this case the entropy of the eigenvalues is minimal (close to 0), consequently S is close to 1, meaning maximal contraction of the embedding dimension, namely strong synchronization.

Due to its mathematical construction, the two extremes of the S estimator have also the meaning of high entropy (maximum activity dispersion) for S = 0 and low entropy (minimum activity dispersion) for S = 1. Furthermore, we computed the difference (trial by trial because of the known temporal order) ΔS = S_BKG_-S_stimulus_, where its positive values denote decreased synchronization with respect to the background, and negative values indicate increased synchronization. To assess statistical changes in the degree of synchronization, we used non-parametric tests because S values are restricted between 0 and 1 and, consequently, the hypothesis of Gaussian distribution is not tenable. We used Wilcoxon signed rank test to test the null hypothesis of zero median for the ΔS = S_BKG_-S_stimulus_; we used Wilcoxon rank sum test to assess the null hypothesis of equal medians for ΔS computed in two different conditions (control and cooling).

To assess phase synchronization we used the modulus of the Order Parameter [33], a measure sensitive only to coherence in the phase and not in the amplitude of the signals. The Order Parameter may be interpreted as the collective rhythm produced by the whole population of system's phases under study. For instance, if all systems move in a single tight clump this measure is approximately 1 and the population acts like a giant oscillator. If the individual oscillations add incoherently and no macroscopic rhythm is produced, this measure is approximately 0. It corresponds to the centroid of the phases extracted from the signals. The separation of phase and amplitude was achieved by employing the analytical signal concept, i.e. by means of the Hilbert transform [34].
